# New Gel Approaches for the Transdermal Delivery of Meloxicam

**DOI:** 10.3390/gels11070500

**Published:** 2025-06-26

**Authors:** Ioana-Alexandra Plugariu, Maria Bercea, Luiza Madalina Gradinaru

**Affiliations:** “Petru Poni” Institute of Macromolecular Chemistry, 41-A Grigore Ghica Voda Alley, 700487 Iasi, Romania; bercea@icmpp.ro (M.B.); gradinaru.luiza@icmpp.ro (L.M.G.)

**Keywords:** gel, meloxicam, non-steroidal anti-inflammatory drug, transdermal delivery, absorption promoters

## Abstract

Meloxicam is a promising non-steroidal anti-inflammatory drug (NSAID) for acute and chronic pain prevention and treatment. Due to its poor water solubility, the clinical use of meloxicam is limited. In addition, for transdermal applications, the impermeability of the skin makes it difficult to conceive an appropriate NSAID-based delivery system that can penetrate through the skin barrier. Hydrophilic/hydrophobic gels, designed as transdermal drug delivery systems, can considerably improve other drug administration types (such as oral or intravenous), avoiding or limiting the side effects. The main purpose of this paper is to present some physicochemical and pharmaceutical considerations about meloxicam and to review the most important research concerning the gels used for the transdermal delivery of meloxicam. Thus, smart polymeric networks, semi-solid systems (lipogels, emulgels), β-cyclodextrin-based gels, liposomes (ethosomes, niosomes, flexosomes, transferosomes, menthosomes, invasomes), and nanostructured lipid carriers, with analgesic and anti-inflammatory activity, are discussed. The key objective of this study was to highlight various gel formulations with enhanced properties, which could be used in a minimally invasive manner for the sustained administration of meloxicam.

## 1. Introduction

Transdermal drug delivery systems have received great interest in the pharmaceutical field due to their potential benefits over traditional methods, including oral or injectable delivery [1,2,3,4,5,6,7,8]. The skin is permeable to small molecules and lipophilic drugs, but it is impermeable to polymer chains or hydrophilic drugs. Drug diffusion across the skin can be drastically limited by the stratum corneum (the outermost layer of the epidermis). Regenerative medicine investigates the possibility of overcoming skin resistance to reduce pain and accelerate wound healing. Ignoring inflammation is a primary reason for pain and the damage of tissues. One possibility to overcome these difficulties is the use of the non-steroidal anti-inflammatory drugs (NSAIDs). The proper use of medication can minimize distress and significantly enhance the quality of life.

Meloxicam (MX) is one of the compounds belonging to the enolic acid class of NSAIDs that preferentially inhibits cyclooxygenase-2 isoform (COX-2) over cyclooxygenase-1 (COX-1). MX is remarkable for its effective analgesic, antipyretic, and anti-inflammatory properties [1,6,7,8,9,10,11,12,13,14]. MX is an active principle of pharmaceutical interest, with a minimum therapeutic effective dose (that is lower than that of the majority of NSAIDs) and with limited side effects [15,16]. Although the solubility of MX in water is very low [9,17], many efforts have been carried out to incorporate it into appropriate matrices due to its analgesic and anti-inflammatory activity. Various forms of gels seem to be suitable solutions to load and deliver this active principle for both human [18,19,20] and veterinary use [21,22].

Orally administered MX-based pharmaceuticals may have adverse effects on the gastrointestinal tract and even reduce the lifespan of patients with rheumatoid arthritis. Transdermally delivered MX ensures consistent plasma levels from a single dose, prevents significant gastrointestinal side effects, and enhances local analgesia [7]. The best strategy for improving drug penetration into the skin is to choose suitable vehicles with the optimum characteristics.

In this regard, the current review provides an overview of the studies carried out to obtain improved gel formulations, with prolonged MX release that are applied in a minimally invasive way. Stimuli-sensitive gels, modern vesicular delivery systems, as well as nanostructured lipid carriers are briefly discussed. Furthermore, the physicochemical and pharmaceutical properties of MX, as well as the risks and benefits of co-administering MX with other active substances, are also highlighted.

## 2. Physicochemical Properties and Pharmaceutical Profile of Meloxicam

### 2.1. Chemical Structure

**Scheme 1:**
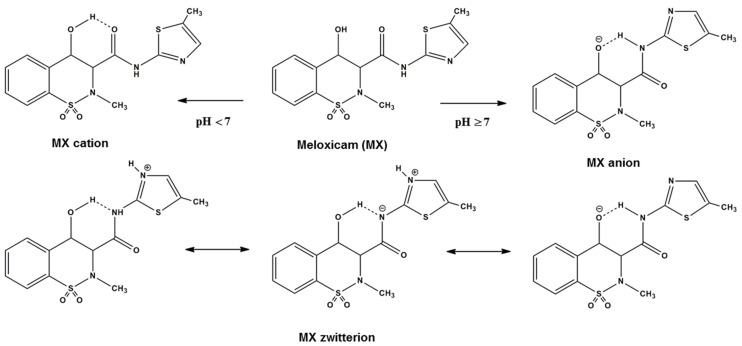
Tautomer forms of MX: cation, anion, enol, and zwitterion. Adapted with permission from [9] (copyright: 1996, Elsevier).

Meloxicam (MX) is an active substance that belongs to the oxicam class, with the structural formula C_14_H_13_N_3_O_4_S_2_ and IUPAC nomenclature 4-hydroxy-2-methyl-N-(5-methyl-2-thiazolin)-2H-1,2-benzothiazian-3-carboxamide-1,1-dioxide [12,20]. Depending on the pH values and the polarity of the solvent, MX exists in different tautomer forms [9] (Scheme 1).

For example, at neutral pH, the anionic form is predominant. Most often, polar solvents favor the formation of the zwitterion form of MX, while non-polar solvents lead to enolic forms. On the other hand, the acidic pH favors the formation of the cationic structure [9,23].

### 2.2. Solubility

MX is insoluble in water and slightly soluble in propylene glycol (PG) or liquid paraffin. Surfactants, such as Span 20 and Tween 20, enhance MX solubility [24].

In reaction with bases, MX forms salts, increasing the solubility of the active substance in administration conditions. For example, with ammonia or sodium hydroxide, it forms ammonium and sodium salts, respectively. An acidic pH is unfavorable for MX incorporation, as it becomes difficult to dissolve it in an aqueous or an alcohol-based environment. Also, in its enolic form, MX can be recrystallized from tetrahydrofuran. The zwitterion structural form results by dissolving MX in an aqueous sodium hydroxide solution, whereas the cationic structure predominates at a low pH [9,23].

The solubility of NSAIDs and the ratio of ionized to non-ionized forms are responsible for the occurrence of adverse gastrointestinal reactions, depending on how much the active substance is reabsorbed from the gastrointestinal tract. A low pH favors the transition from the non-ionized to the ionized form, leading to a decrease in the solubility of the active substance.

**Figure 1:**
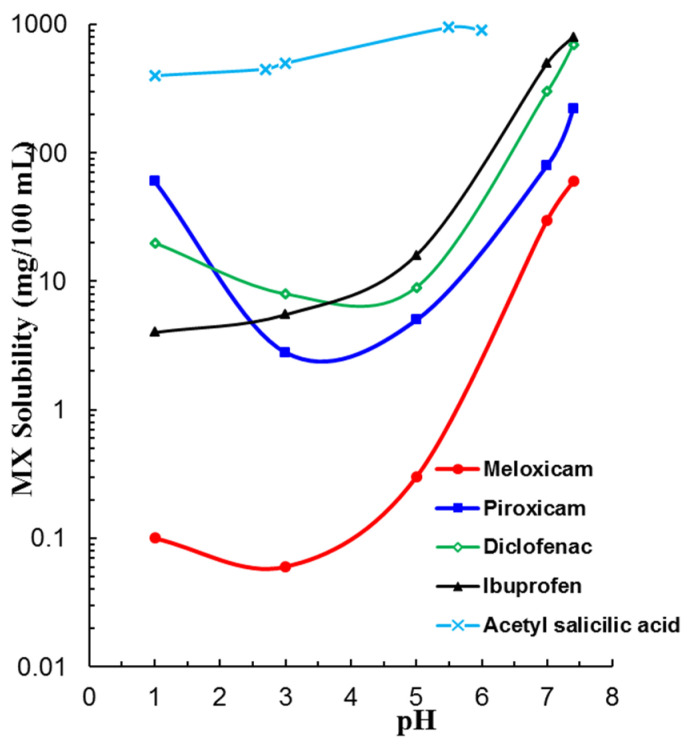
Representation of the solubility of different NSAIDs as a function of pH. Adapted with permission from [9] (copyright: 1996, Elsevier).

By using HPLC methods, the solubility of MX was highlighted in relation to other NSAIDs, such as piroxicam, diclofenac sodium, ibuprofen, and acetyl salicylic acid (Figure 1) [9]. According to this representation, acetyl salicylic acid appears as the most soluble NSAID, even in an acidic environment. Most of its molecules exist in the non-ionized form that easily crosses the gastric membrane and can induce negative effects [25]. On the other hand, MX is the NSAID in this class that is most challenging to dissolve [9].

The MX solubility influences the loading ability, which is a key factor in developing new therapeutic gels: a higher drug loading can significantly improve the pharmaceutical formulation.

### 2.3. Therapeutic Indications and Contraindications

The therapeutic efficiency is due to the ability of NSAIDs to reduce pain, fever, and inflammation, being indicated in conditions such as rheumatoid arthritis, seronegative spondyloarthritis, osteoarthritis, postoperative pain, and osteochondrosis [26,27]. MX is very versatile in a multitude of conditions, especially in inflammatory, rheumatic, and bone diseases [12,27]. MX is indicated for reducing pain caused by chronic conditions. This active substance can also be used to treat various inflammatory conditions, as well as degenerative joint diseases, in which pain is the main reason for discomfort [14,27]. Moreover, MX is considered the active substance of choice in osteoarthritis as it promotes the synthesis of proteoglycans [28,29] and it is used especially for associated symptoms like inflammation and pain. Another indication of MX is lower back pain syndrome [30]. On the other hand, NSAID administration is associated with adverse gastrointestinal effects, such as gastropathy, which decrease the patient’s compliance with the treatment [31].

The gastrointestinal toxicity of MX has been compared with that of other NSAIDs. Several studies involving over 5600 patients have demonstrated that MX exhibits significantly lower gastrointestinal toxicity compared to naproxen, diclofenac, and piroxicam [9,32,33]. Compared to diclofenac, MX is associated with fewer adverse effects, such as dyspepsia, nausea, vomiting, abdominal pain, and diarrhea [32]. Additionally, MX showed a lower incidence of thromboembolic effects than diclofenac [34].

A long term study was conducted involving 357 patients to which 15 mg/day MX was administered over 18 months [35]. Thus, MX was compared to other NSAIDs regarding efficiency and tolerance. The most common adverse effects were gastrointestinal, musculoskeletal, skin, and respiratory issues [36]. Also, compared to naproxen, MX has a lower incidence of gastrointestinal side effects. It is generally better tolerated [37]. Another aspect is that NSAIDs are contraindicated for patients with pre-existing gastrointestinal conditions [38] and for patients suffering from comorbidities like hepatic cirrhosis, congestive heart failure, nephrosis, etc. In the final group of patients, the use of NSAIDs raises the risk of developing acute renal failure [39]. On the other hand, administering MX in the maximum allowed doses was not associated with the impairment of renal function [40]. Unlike other NSAIDs, MX does not influence platelet aggregation and bleeding time [41].

### 2.4. Pharmacokinetic Profile of Meloxicam

The intravenous administration of MX determines a plasma concentration of 3.7 µg/mL in 12 h, which was similar to intramuscular administration [42]. MX has a bioavailability of 89% [26], and most of the active substance binds to plasma proteins (approx. 90%) [35,43]. From a pharmacokinetic point of view, MX has a long half-life of 20–24 h [14]. Additionally, MX can reach therapeutic concentrations in the synovial fluid. The active substance appears within one hour, and maximum concentration is attained six hours after administration [44]. The pharmacokinetic profile of MX is not notably affected in vulnerable patient groups, such as the elderly and those with mild kidney or liver conditions. The intramuscular route offers a quicker response than the oral route, where the gastrointestinal passage delays absorption. This is especially useful in cases requiring rapid intervention to suppress some symptoms quickly, such as acute pain, or when the patient’s condition necessitates this method of administration [26,40]. Moreover, by intramuscular administration, the active substance can exert its therapeutic effect in 1.5 h, having 100% bioavailability. The biotransformation of MX primarily occurs in the liver, with excretion taking place in the biliary and urinary systems [40].

The pharmacokinetic profiles of the active substances can influence the pharmacological response’s speed. Thus, in a double-blind study, the effect of MX on the reduction in periarticular shoulder pain was investigated compared to piroxicam. Both active substances were administered orally in doses of 7.5 or 15 mg/day and piroxicam 20 mg/day. Pain relief was monitored over a period of 7 days. The results indicated a quicker response to MX administration than piroxicam. This can be attributed to the different pharmacokinetic profiles of the two substances (MX reaches the maximum plasma concentration quickly) [45].

### 2.5. Pharmacodynamics of Meloxicam

The mechanism of action of NSAIDs consists of the inhibition of the cyclooxygenase enzyme (COX) with a reduction in the synthesis of prostaglandins involved in the inflammatory process. COX represents a multienzyme complex containing the isoforms constitutive COX-1 and inducible COX-2. Unlike COX-2, which promotes inflammation, fever, and pain, COX-1 ensures the protection of the mucous membranes and different functions at the renal level, such as glomerular filtration, renin secretion, renal blood flow, etc. [46]. The occurrence of adverse reactions is correlated with the inhibition of the COX-1 isoform. It has the role of protecting the stomach lining through prostaglandins. Inhibiting this enzyme selectively reduces adverse gastrointestinal effects, such as epithelial erosion and bleeding [47].

MX can selectively inhibit COX-2 [22]. It has been proved that MX has a wide range of safety due to its predominant COX-2 inhibitory action [14,48]. Thus, in a study involving 76 healthy volunteers, the selective COX-2 inhibition by MX was compared with that of rofecoxib (a selective COX-2 inhibitor) [48]. MX demonstrated a higher level of COX-1 inhibition than rofecoxib, but it was similar to diclofenac and lower than ibuprofen and naproxen. MX exhibits a stronger affinity for COX-2 than COX-1 [48].

Another study examined the effectiveness of MX in inhibiting eicosanoid production, which are lipids synthesized through the cyclooxygenase pathway. The results were compared to those of indomethacin, with samples collected from patients suffering from both malignant and benign gastric diseases in a suitable culture medium [49].

The inhibition of COX-2 is associated with the suppression of pain and inflammation [1,44,50]. Also, MX is an active substance with anti-inflammatory effect. It is also due to its ability to inhibit oxidative phosphorylation, neutralize active oxygen species that favor the inflammatory process, and reduce inflammation-specific mediators, such as proteinases, platelet activation factors, etc. [14].

Therefore, the lower inhibition of both COX-1 compared to indomethacin and COX-2 compared to rofecoxib suggests an optimal therapeutic profile. The pharmacodynamic profile of MX offers advantages over other compounds, including lower cardiovascular risks and better gastric tolerance [26,50].

### 2.6. Pharmacography

The most commonly used formulations of MX include tablets, injectable solutions, suppositories, and topical pharmaceutical forms (e.g., 1% gel). In Europe, topical preparations that contain MX are unavailable, as this active substance is difficult to incorporate. MX is hardly soluble in water and most solvents [9]. Topical formulations offer several benefits such as preventing gastrointestinal absorption, targeting a specific body area for therapeutic effects, increasing patient compliance, being easier to apply, and often being preferred especially for chronic conditions [27].

New formulations should be optimized to ensure the patient’s compliance with the treatment. In this regard, specific conditions must be fulfilled, including tissue adhesion, ease of use, and therapeutic efficacy [51].

### 2.7. Synergistic Effects of Meloxicam and Other Active Substances

The interaction of MX with other active substances was investigated in order to enhance the anti-inflammatory effect, reduce adverse reactions, or improve formulations and other therapeutic actions. Thus, MX can be combined with other active ingredients, such as antibiotics, anesthetics, antiseptics, and more, to broaden the action spectrum or reduce specific symptoms in certain conditions. Additionally, MX can be combined with other plant-based therapeutic agents to enhance its anti-inflammatory effects or reduce the required dose. Thus, adverse reactions are limited by using the minimum effective concentration of MX. On the other hand, plant active principles can function as a phytocomplex, like essential oils. They can act through specific therapeutic properties, increasing the bioavailability of the active substance at the site of action. Therefore, essential oils can be seen as enhancers of MX, facilitating the crossing of the epithelium and improving the therapeutic effect [19]. An example of this is Eucalyptus essential oil (*Eucalyptus globulus*). The active principle is eucalyptol (1,8-cineole), which has demonstrated analgesic and anti-inflammatory properties [52,53]. Eucalyptol enhanced bioavailability and therapeutic activity [17,19].

The pharmacokinetic profile of MX is not changed by antacids or cimetidine. Additionally, MX does not influence the pharmacokinetics of other concurrently administered drugs, such as digoxin, furosemide, or methotrexate. The administration of warfarin simultaneously with MX should be conducted with caution, as NSAIDs can enhance the effects of this active substance [40].

MX can be administered in combination with bupivacaine to induce an analgesic/anti-inflammatory effect and to obtain extended-release systems. Small doses of MX are sufficient to potentiate the analgesic properties of bupivacaine [4,54,55]. Additionally, MX can be formulated with rubefacients. These have the role of increasing vascular dilation and are considered absorption promoters, as they have a beneficial effect on the absorption of the active substance. These include capsaicin, methyl salicylate, camphor, menthol, and isopropanol [56,57].

### 2.8. Meloxicam’s Protective Role in Cardiovascular Health

Multiple experiments and clinical studies have shown that MX is as effective as selective NSAIDs while offering an improved safety profile [34]. It is advantageous due to its good tolerance for long-term use and it is suitable for treating chronic conditions [27]. On the other hand, MX demonstrated low cardiovascular risk [50,58]. The anti-inflammatory and analgesic activities are potentiated by COX-2 inhibition, as previously discussed, but at the same time, the cardiovascular risks also increase [50]. In this sense, the risk/benefit balance must be monitored. MX administration demonstrates a beneficial effect on the cardiovascular system by reducing vascular inflammation [58]. MX has an optimal therapeutic and safety profile, being a preferred option among NSAIDs [40]. Thus, the active substance MX can be used in topical formulations as a therapeutic option, especially for local inflammations.

## 3. Gel Formulations Featuring Meloxicam as Active Compounds

The physicochemical profile of MX makes it suitable for topical formulations. Thus, properties such as the ability to easily cross the lipid skin layer, low molecular weight (354.1 g), high potency at low doses, and lack of skin toxicity are favorable for the skin administration of MX [59]. On the other hand, the local administration of NSAIDs offers the advantage of bypassing hepatic metabolism and minimizing gastrointestinal side effects [60]. MX can be considered a potential therapeutic option for treating conditions that require localized anti-inflammatory effects. Thus, its incorporation into different gels becomes challenging due to the specific insolubility of MX in an aqueous environment. The MX release profile is influenced by the interactions between the vehicle and the drug molecules. The transdermal absorption of active molecules depends on the release rate, the skin permeability, and the gel viscosity.

### 3.1. Smart Polymer Networks

In situ gelling systems incorporating active substances represent suitable vehicles for delivering drugs with low solubility in water [61,62,63,64,65]. These systems showed liquid-like behavior below the lower critical solution temperature (LCST); above LCST, micelles with a hydrophilic core and a hydrophobic shell are formed. As the polymer concentration and temperature increase, the hydrophobic interactions lead to a sharp sol–gel transition [17,64,65]. The poor solubility of MX in the aqueous environment is the main difficulty in its incorporation into delivery vehicles. Many efforts have been oriented toward suitable ways to potentiate MX transdermal delivery and promote local analgesia, minimizing the side effects [7,8,28,29].

Poloxamers (also known as Pluronics) are water-soluble triblock copolymers made of two hydrophilic poly(ethylene oxide) blocks and one central hydrophobic poly(propylene oxide) block. At physiological temperature, poloxamers in aqueous solutions (usually concentration higher than 15%) spontaneously generate networks formed by polymicelles. These thermoresponsive systems are excellent excipients for incorporating various hydrophilic and hydrophobic active principles, being of high interest for injectable gels in tissue engineering or drug delivery applications [64,65,66,67]. However, poloxamer gels lose their structural integrity within several days upon prolonged exposure to water or other solvents [68]. Polysaccharide addition to poloxamer gels was considered a possibility to improve the biological and rheological properties, ensuring shape fidelity and gel stabilization against dissolution [3,62,68,69,70]. The composite gels are stable in an excess of water or biological fluid [68] and present improved mechanical properties as compared with pure poloxamer networks [70]. Poloxamer formulations supplemented with various stabilizing agents are suitable as viable cell encapsulation materials. Cell viability was improved by adding hydrocortisone, glucose, or glycerol as stabilizing agents [71].

Generally, the MX content (as active substance) in various gels is 1%. The release of the active substance occurs most rapidly from the gel formulations, compared to ointments or creams [72]. The in vitro release of this drug through a semipermeable membrane in comparison with rat skin was investigated using different polymers as vehicles for the active principle: methyl cellulose (MC), hydroxyethyl cellulose (HEC), poly (vinyl alcohol) (PVA), poloxamer 407 (P407), poly (ethylene glycol) (PEG), and Carbopol 974P. The effect of the initial drug concentration and polymer content on the release rate and anti-inflammatory activity was evidenced. The fastest release of MX was observed for the formulation containing MC, followed by HEC, P407, and PVA [73]. This could be attributed to the interaction between the active ingredient and the polymer, which depends on the nature and structure of the vehicle [74].

Another study used different vehicles, including carbomer, xanthan gum, carmellose sodium, and hypromellose were used to prepare anti-inflammatory gels. Among the excipients, propylene glycol (PG) served as a humectant, while sodium benzoate acted as a preservative. Triethanolamine (TEA) was also added to the carbomer gel as an absorption promoter. The formulation that facilitates the rapid release of MX is the one containing Carbopol, while the formulation with hydroxypropyl methyl cellulose (HPMC) exhibits the slowest release [21].

Furthermore, absorption promoters are essential in topical formulations, as they enhance the absorption of the active substance, improve bioavailability, and favor an optimal pharmacodynamic response. Fatty acids or alcohols can be mentioned as absorption promoters [75]. Thus, different absorption promoters, including ethanol, PG, menthol, and azones, were compared in the study and the ability of these substances to enhance MX absorption was evaluated. The most promising result was obtained by menthol, followed by azones, ethanol, and PG [76].

Depending on the temperature, thermoreversible gels can change their physical state between solid and liquid [66,77,78]. These gels are very versatile and can be used as systems for the sustained release of active substances, by creating an in situ deposit [79]. MX was incorporated in temperature-sensitive poloxamer-based gels and bupivacaine was also included in the form of multivesicular liposomes as the second active principle. In addition, chitosan and β-glycerophosphate disodium salt hydrate were added to obtain an optimal pH and osmotic pressure [18,80,81]. MX loaded into these temperature-sensitive hydrogels promotes the release of bupivacaine by reducing inflammation and changing the local pH [79].

**Figure 2:**
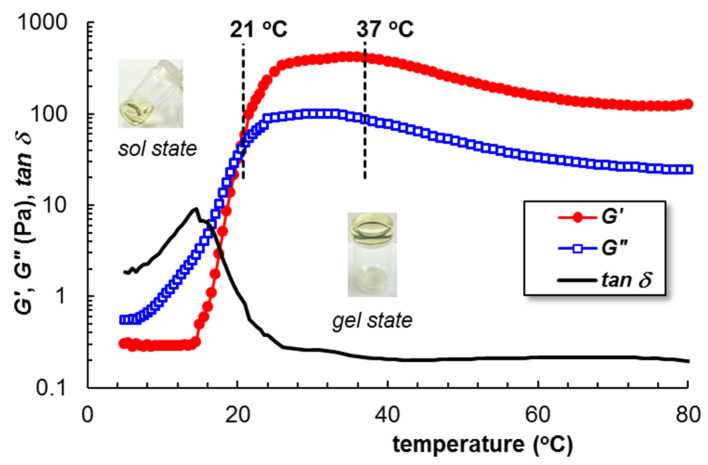
The viscoelastic parameters *G*′, *G*″, and *tan δ* as a function of the temperature for a PU sample loaded with MX and submitted to a heating rate of 0.5 °C/min. The transition from the sol state to the gel state was identified around 21 °C [17].

Polyurethanes (PUs) represent another category of polymers able to form thermoreversible gels [82,83,84,85]. Thus, at low temperatures, they exist as individual entities (macromolecules, micelles) dispersed in the solution state and, under physiological conditions, they become tridimensional networks (Figure 2). These polymers have an amphiphilic character, which is advantageous for most poorly soluble active substances. The resulting gels are thermoreversible, can be administered parenterally, and demonstrate sustained release [17,84,85,86,87,88].

**Figure 3:**
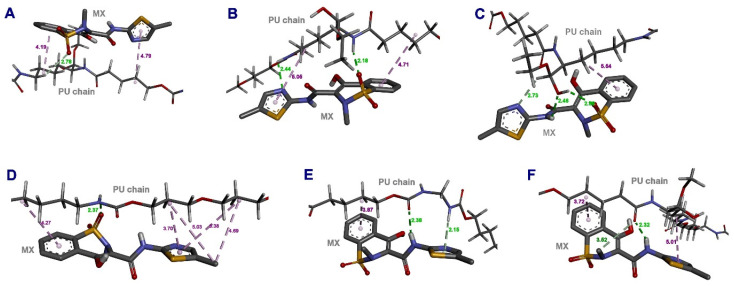
Docking model showing the most favorable interactions of MX with the PU matrix: (**A**–**C**) involving the sulfone group of MX; (**D**–**F**) mainly hydrophobic interactions in MX at the level of aromatic rings. The two compounds are shown in the stick representation. Color code: magenta—hydrophobic interactions; green—hydrogen bonding. The distance between the donor and acceptor is indicated in Å [17].

In the presence of MX, the interactions between the urethane groups and the ether groups from PU are changed. A stable PU–MX matrix was identified by molecular docking (Figure 3), with the binding affinity around −3 kcal/mol [17]. The urethane groups located in the interphase are accessible for interactions with the sulfone groups of MX molecules (Figure 3A–C). When MX was introduced into the PU matrix in the sol phase, some intramolecular interactions between functional groups of the PU macromolecule were broken and new intermolecular interactions between PU chains and MX were established. Also, the competition between the hydrophobic interactions and hydrogen bonding will dictate the network stability (Figure 3D–F).

An important feature of thermosensitive hydrogels is the gradual release of the active substance, which is advantageous in chronic conditions that require administration over an extended period of time [89,90]. Different excipients were introduced into the gel formulations to improve the absorption of MX and to achieve sustained release: PEG, poly(vinyl pyrrolidone) (PVP), carboxymethyl cellulose (CMC), and hydroxypropyl cellulose (HPC) [12,91,92,93]. Incorporating various principles can impart biological properties to the hydrogel, such as antimicrobial, anti-inflammatory, antioxidant, etc. For example, in this case, oregano essential oil was added, which produced a gel with antimicrobial properties [17,94].

**Figure 4:**
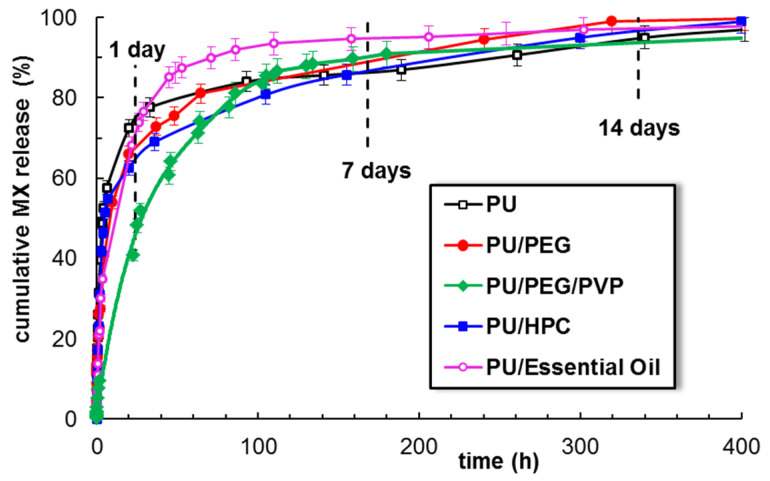
Meloxicam release kinetics from 25% PU hydrogel (1) and hydrogel samples containing different excipients: PEG (2) PVP (3), HPC (4 and 5), and *Oregano Compactum* essential oil (6). Adapted from [17].

The beneficial effect of essential oils added to PU gels was recently revealed [17]. Figure 4 shows the fast release of MX from hydrogels containing only PU or PU in the presence of 3% *Oregano Compactum* essential oil. A prolonged release of the active substance was demonstrated in the presence of various excipients, such as 3% PEG, 3% PVP, and 6.25% HPC.

### 3.2. Lipogels

Lipogels are lipid-coated polymeric gels with a hydrophilic core and a lipophilic shell, able of trapping both hydrophilic and hydrophobic molecules.

**Figure 5:**
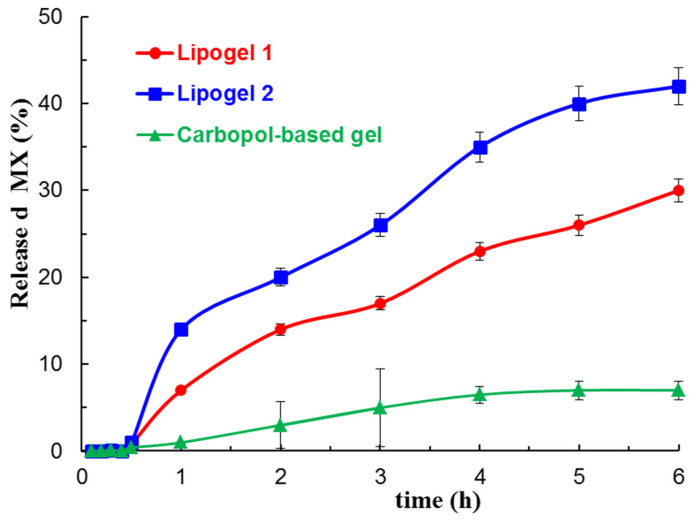
Meloxicam release from Lipogel 1, Lipogel 2, and Carbopol-based gel using a mixed cellulose esters membrane in a Franz diffusion cell. Adapted with permission from [95] (copyright: 2007, Elsevier).

The delivery of MX from olive oil-based lipogels, containing ethyl cellulose (EC) and olive oil-derived products (such as PEG-4 olivate, denoted Lipogel 1, or Sorbitan olivate, denoted Lipogel 2), was investigated in comparison with a Carbopol hydrophilic gel containing TEA and PG [95]. The diffusion of the active substance was improved using different Millipore membranes. Lipogels offer the advantage of enhanced skin absorption due to their capacity to penetrate the stratum corneum. A faster release of MX was observed from Lipogel 2 (Figure 5), attributed to a lower affinity between the vehicle and the active principle. On the other hand, for the Carbopol-based formulation, the stronger affinity between MX and the vehicle results in a slower release of the active ingredient. For similar shear stress values, the rheological characteristics (especially the viscosity value) were more favorable in the case of Lipogel 2 [95].

### 3.3. Emulgels

**Figure 6:**
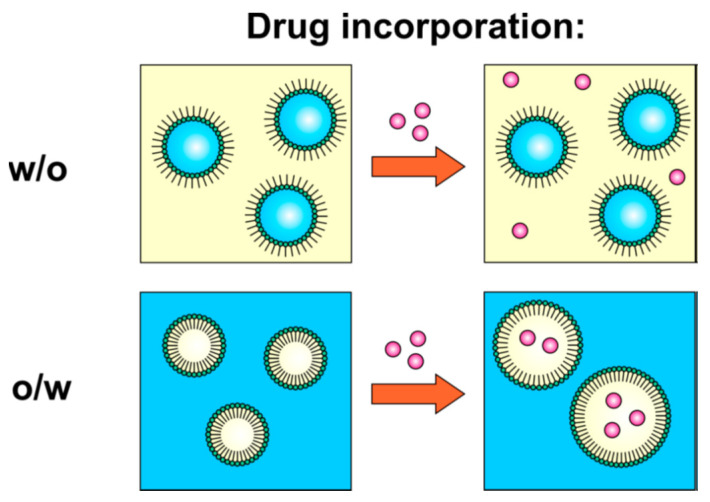
The distribution of the active substance in w/o or o/w microemulsions [96].

Emulgels are amphiphilic networks able to control the release of drugs presenting different polarities. Usually, emulsions are thermodynamically stable systems composed of polar and non-polar phases, such as water/oil (w/o) or oil/water (o/w) dispersions (Figure 6). The emulgels combine hydrophilic surfactants with high values of hydrophilic–lipophilic balance (HLB) with lipophilic cosurfactants with low HLB values [96]. The presence of polymer chains in the hydrophilic region leads to thermodynamically stable systems.

Topical emulgels have several benefits, such as increased patient compliance, thixotropic characteristics, and spreadability. The formulations of MX-based emulgels have proven optimal clinical results in osteoarticular diseases [97,98].

The ability to relieve pain and inflammation with topical emulgel is comparable to oral or intravenous formulations. Emulgels are more stable than lipogel formulations and demonstrate superior absorption. This is due to the greater solubility of MX in surfactants compared to the lipophilic phase [12].

The release kinetics of MX from formulations containing different concentrations of carbopol and menthol were studied. Menthol is a promoter of absorption and favors the release of the active substance. The emulgel is formulated by adding the surfactants tween-20 and span-20 and a penetration enhancer, capsaicin, which increases the bioavailability of MX. It was observed that the optimal formulations were those containing the lowest concentrations of Carbopol (0.5%) and the highest concentrations of menthol (9%). This behavior can be attributed to the change in viscosity depending on the polymer concentration [24].

Nanoemulsions are part of the category of nanocarriers, along with ethosomes, niosomes, liposomes, and nanoparticles. These systems favor the optimal penetration and absorption of the active substance [99]. The component particles have sizes ranging from 100 to 500 nm. The formulation is based on a lipophilic–hydrophilic phase, a surfactant, a cosurfactant, and a gelling agent added to increase the viscosity [100,101]. Another advantage of nanoemulgels is their broad scope of applicability, allowing them to exhibit various therapeutic properties, including anti-inflammatory, analgesic, antifungal, antibacterial characteristics, etc. [100]. Thus, caprylic acid was used as lipophilic phase in a nanoemulsion formulation, Tween-80 was used as surfactant, PG was the co-surfactant, and Carbopol functioned as the polymer matrix. This formulation demonstrated anti-inflammatory activity against rat paw edema and promoted an optimal concentration of the active substance at the site of action [102]. Caprylic acid-based nanogels ensure the optimal release of MX; they are biocompatible, non-toxic, and characterized by Newtonian flow and good spreadability [102].

### 3.4. β-Cyclodextrin-Based Gel Formulations

Using cyclodextrins to incorporate poorly soluble substances has numerous advantages, such as enhanced solubility, improved absorption, increased stability of the active substance, and reduced adverse reactions [103,104]. Cyclodextrins exhibit an amphiphilic character, being able to incorporate poorly soluble medicinal substances [105]. MX loaded into β-cyclodextrin (β-CD)-based gels were obtained using different polysaccharides: cellulose, sodium alginate, HPMC, CMC, chitosan, dextran, hyaluronic acid, guar gum, gellan gum, xanthan gum, pectin, carrageenan, or starch [106]. The release of MX from these complex gels is influenced by the concentration of the gel base and the nature of the polysaccharide. The sample containing HPC exhibits the fastest release, favored by the increase in polymer concentration [107].

The formulations presented in the literature also contain absorption promoters such as menthol, thymol, cineole, oleic acid, or PEG. Cineole at a concentration of 5% increased the release rate of MX. On the other hand, a higher concentration of cineole (for example 10%) decreased the release of the active substance. Another absorption promoter, menthol, used at 5% concentration, increased the bioavailability of the active substance at the site of action. It acts through two mechanisms: firstly, it forms a eutectic mixture with the active substance, and then, it interacts with the phospholipid barrier [108,109].

Also, thymol provides the most effective release of MX, but the resulting gels are brittle [110]. On the other hand, 1% oleic acid acts as an absorption promoter that enhances the release of MX. The resulting gels are optimal, stable, and consistent [111].

A reduced release of MX from the formulations based on cyclodextrins and chitosan was observed. This can be explained by the interaction between chitosan and the inclusion complex [112]. However, in other formulations, an improvement in MX release is observed, due to the formation of a MX- β-CD complex. Thus, the release of MX is increased in formulations containing CMC, HPC, and β-CD. These polymers are water-soluble and favor the formation of a complex between the MX and β-CD [113]. The HPMC gel containing the β-CD complex released MX the fastest. In these formulations, cineole and menthol were found to be the most effective absorption promoters [114]. Additionally, compounds derived from β-CD, such as hydroxypropyl-β-cyclodextrin (HP-β-CD), functioned as permeation enhancers, facilitating the rapid absorption of the active substance [115]. Thus, formulations based on HP-β-CD have demonstrated an increased release of active principles compared to β-CD, resulting from the improved solubility and dissolution of substances, with a faster onset of the targeted pharmacological effect [116]. Moreover, these formulations enhance the solubility, stability, and bioavailability of the active substance at the site of action [117].

### 3.5. Modern Vesicular Delivery Systems

#### 3.5.1. Liposomes

**Figure 7:**
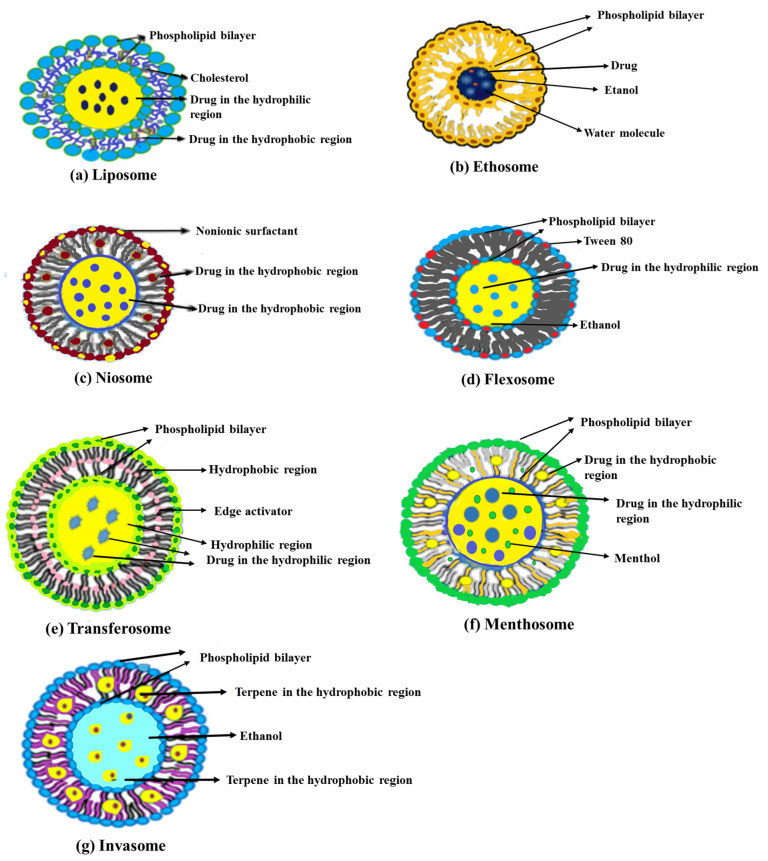
Schematical presentation of several types of vesicular systems: (**a**) liposome; (**b**) ethosome; (**c**) niosome; (**d**) flexosome; (**e**) transferosome; (**f**) menthosome; (**g**) invasome. The schemes (**a**–**g**) were adapted with permission from [126] (copyright: 2021, Elsevier).

Liposomes are spherical structures characterized by their amphiphilic nature. They consist of one or more layers of phospholipids [118]. Liposomes are of interest due to some advantages, including low toxicity, biocompatibility, amphiphilic character favorable for most poorly soluble substances, and the possibility of adding absorption promoters [119]. However, liposomal formulations have some drawbacks, including instability and low patient compliance due to high cost [120,121]. The amphiphilic nature of liposomes enables the incorporation of poorly soluble active substances. However, the penetration of active substances into the skin occurs only in the superficial layers [118]. The type of phospholipid layers, the preparation method, the composition, and the size of the vesicles determine the degree of flexibility or rigidity [122,123,124,125]. Deformable liposomes were introduced to enhance the bioavailability of the active substance, achieving therapeutic concentrations of the active principle in the deepest layers of the skin. In this regard, flexible membrane vesicles have been developed, including ethosomes, niosomes, flexosomes, transfersomes, menthosomes, and invasomes (Figure 7, Table 1), which can easily cross the skin’s phospholipid layer. Also, vesicular systems for the controlled release of active principles are numerous, including pharmacosomes, herbosomes, sphinosomes, herbosomes, and exosomes [126]. Exosomes are of natural origin, having important advantages, such as high biocompatibility and therapeutic efficacy [127]. On the other hand, deformable liposomes are based on phospholipids, a penetration enhancer, and an edge activator [128,129].

Liposomal formulations have some drawbacks, such as their large size and rigidity (which limit their access to deeper areas of the skin) [130,131], physical instability, easy degradation, and instability during storage [132]. Furthermore, it is challenging to obtain these systems on a large scale and to achieve sterilization with them [133]. Certain well-defined production methods (extrusion, thin film hydration) are reproducible, but meloxicam delivery systems have low scalability (Table 1).

A detailed discussion of the advantages and disadvantages of vesicular systems was presented by Singh et. al. [137]. Generally, there is a high gap in their preclinical tests and clinical translation [129,131,135,139,142,143,144,145].

#### 3.5.2. Ethosome Gel Formulations

One of the criteria for an optimal topical formulation is that it easily crosses the phospholipid barrier [146,147,148]. In this regard, ethosomes, which are multilayer vesicles that contain high concentrations of ethanol (Figure 7b), could ensure the optimal bioavailability of the active substances. They facilitate easier passage through the phospholipid bilayer [118]. It has been shown that ethosomes allow for a deeper penetration of the active substance and enhanced delivery [118,149]. In addition, these systems composed of phospholipids, ethanol, and water have the advantages of small size and improved flexibility [150,151,152]. Also, ethosome-based formulations have characteristics such as biodegradability, biocompatibility, non-immunogenicity [153], and protective actions on the phospholipid layer [154].

Thus, in vitro studies have demonstrated a faster MX release from formulations containing ethosomes. This is due to their flexibility and the maintenance of structural integrity [155,156]. Also, the ethanol concentration in ethosomes must have an intermediate value to favor the release of the drug molecules [157,158]. For such systems, standard production methods ensure high scalability.

#### 3.5.3. Niosome Gel Formulations

Niosomes (non-ionic surfactant vesicles) present closed bilayer structures, analogous to liposomes (Figure 7c), from non-ionic amphiphiles by self-assembling in aqueous environments. Such structures can encapsulate solutes, being stable, and osmotically active [6]. Niosome-based formulations have the advantage of penetrating the active substance into the deeper layer of the skin and of a prolonged release of the active substance. Also, these formulations are stable and represent the right solution for poorly soluble active substances [20] and do not require any specific storage conditions [159]. Also, proniosomes, precursors to niosomes, represent a suitable vehicle for the release of active principles. They are more stable than niosomes but must be reconstituted before administration [119].

In a series of papers, niosome-based formulations were reported, and different vehicles such as poloxamers, chitosan, and Carbopol were added. It was found that the gels containing poloxamers released MX faster than those containing chitosan or Carbopol. The release of MX is closely related to the gel’s viscosity and the interactions between the active substance and the vehicle [6,61,160]. Also, it has been observed that, by adding Span 60 as excipient, the release of the active substance increases [161]. Regarding the anti-inflammatory effect, the formulation with poloxamers exhibited the most significant inhibitory effect, followed by the formulations with chitosan and Carbopol. Thus, niosomal poloxamer gels demonstrated the greatest inhibition on the paw of the rats [6]. In another study [20], niosomal gels with MX were prepared. Different polymers were used as vehicles, including sodium alginate, sodium CMC, and HPMC. Various excipients, such as PG, glycerin, and surfactants (Span 60, 65, 80, Brij 35, 58 and Myrj52), have been used. In these topical formulations, glycols and glycerin were added as absorption promoters. The fastest release is obtained by the sample containing CMC as a vehicle, followed by sodium alginate and HPC. The permeability coefficient (*p* < 0.05) suggests that niosome formulations ensure optimal bioavailability and active substance penetration into deeper skin layers.

Gels containing only MX release the active substance more quickly than niosomal systems [133]. Niosomes favor crossing the skin’s phospholipid layer by acting on the stratum corneum. This results in the easy crossing of the skin and a higher concentration of the active substance at the site of action [162,163]. Niosomal formulations using the surfactant Span 60 produced smaller vesicle sizes than those utilizing Tween 80. This was evident in the formulation with Span 60, as indicated by the penetration of MX into the deeper layers of the skin [119].

In conclusion, niosomal formulations containing Tween 80, Brij 58, and Myrj 52 showed a faster release of MX than niosomal formulations with Tween 20, Tween 40, and Brij 35. The pharmacodynamic response to administering niosomal formulations is superior to other formulations [20,145].

#### 3.5.4. Flexosome Gel Formulations

Flexosomes represent another category of carrier vesicles for poorly soluble active substances due to their amphiphilic nature, which is favored by the phospholipids in their composition (Figure 7d). The preparation technique consists of the film hydration method. The formulation is based on phospholipids, Tween-80, and ethanol [164]. Unlike liposomes, which cannot penetrate the deeper layers of the skin, flexosomes have the advantage of a flexible membrane [141]. The optimal particle size for easy penetration into the skin should be under 300 nm. Flexosome-based formulations meet these criteria, with particle sizes smaller than those of liposomal formulations [165,166]. Flexosomes facilitate the penetration of the active substance into the deeper layers of the skin. This is evidenced by achieving a 1.5–2.7 times higher concentration of MX in flexosome-based systems compared to MX solutions [167,168]. The surfactant Tween 80 plays an important role in increasing the flexibility of the flexosome membrane [169]. Increasing the ethanol concentration has been shown to enhance the permeation of MX through the skin [152,170,171]. Thus, flexosome-based systems containing phospholipids, a Tween 80 surfactant, ethanol, and a Carbopol-type polymer vehicle can be effective MX delivery systems, ensuring a higher concentration of the active substance and improved bioavailability compared to solution-type MX systems [164].

#### 3.5.5. Transferosome Gel Formulations

Transferosomes are considered the first class of liposomes, characterized by their flexible membrane (Figure 7e). They are based on phospholipids, an edge activator, and surfactant [139,149,172], such as sorbitan esters (Span 60/65/80) and polysorbates (Tween 20/60/80). These compounds enhance the penetration of the active substance through the stratum corneum [173]. Transferosomes have smaller vesicle sizes than liposomes. This is influenced by the presence of an edge activator and the duration and the number of sonication cycles [174,175].

One of the advantages of transferosome formulations is their ability to penetrate MX into the deeper layers of the skin [176]. Transferosome formulations have an active substance bioavailability comparable to subcutaneous administration under specific conditions [149,173,177]. For example, the formulation based on 0.07% MX, 0.04% cholesterol, 0.77% phosphatidylcholine, and 0.1% cetylpyridinium chloride demonstrated the highest bioavailability and the highest penetration coefficient of the active substance through the skin [178]. Compared to microemulsions, transferosomes enhance the penetration of MX to deeper layers of the skin, and unlike liposomes, they tend to accumulate within the skin [138].

#### 3.5.6. Menthosome Gel Formulations

Menthosomes have extremely flexible membranes and are composed of phospholipids, menthol, and an edge activator (Figure 7f) [126,128]. Menthosomes are considered ultradeformable liposomes, with the smallest vesicle sizes and the highest zeta potential values when compared to transferosomes or conventional liposomes [179]. The formulation with the best results was based on 0.077% MX, 0.077% menthol, 0.082% cholesterol, and 0.224% cetylpyridinium chloride [128]. The concentration of cholesterol and phospholipids in menthosomes influences the size of the vesicles and the content of MX [128]. Thus, cholesterol increases the rigidity of the vesicle membrane. In contrast, the presence of a cationic surfactant increases the deformability of the stratum corneum and favors the penetration of MX [180,181]. Additionally, the menthol in the menthosome formulation acts as an absorption promoter. It disrupts the lipid arrangement, enhancing the absorption of MX through the stratum corneum [182].

The concentration of MX achieved after crossing mouse skin was studied. The steady-state flux reached its highest levels with menthosome-based formulations, followed by transferosomes and conventional liposomes [183]. The charge of the vesicle membrane, whether negative or positive, influences the penetration of the active substance through the skin. Menthosomes present a greater positive charge on the vesicle than transferosomes, which will interact with the negative charge of skin, increasing the skin deposit [179].

#### 3.5.7. Invasome Gel Formulations

**Figure 8:**
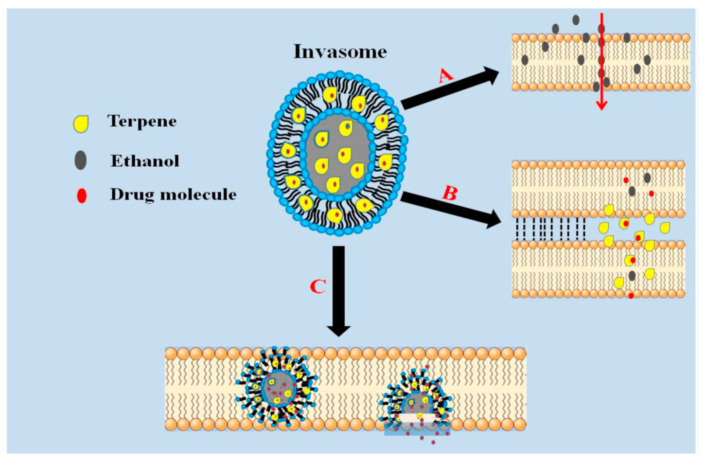
Mechanism of action of invasomes: (A) ethanol-mediated mechanism increases the lipid fluidity and decreases the lipid density. The red arrow indicates the skin penetration; (B) terpene-mediated mechanism breaks transvers hydrogen bonding and widens aqueous region near head groups; (C) vesicle penetration into the lipid membrane. With permission from [126] (copyright: 2021, Elsevier).

Invasomes are vesicular systems composed of phospholipids, ethanol, and terpenes (Figure 7g). These systems achieve a controlled release of the active substance. Still, they have the disadvantage of being unstable over time. They must be stored at a temperature of 4 °C [126,184]. The ethanol and terpenes in the composition act as absorption promoters, facilitating the absorption of the substance through the stratum corneum (Figure 8) [126,185].

In the formulation based on invasomes, terpenes or mixtures of terpenes are used, including limonene, cineole, eugenol, and citral [186]. Terpenes, which are of plant origin, facilitate the absorption of poorly soluble drug substances [187]. Another direct effect observed is that the vesicle pores decrease in size as the concentration of ethanol increases [188]. Terpenes are recognized as absorption enhancers, with cineole being the most effective in facilitating transdermal penetration [184]. Invasomes are delivery systems that ensure therapeutic efficacy by improving the absorption of the active substance and facilitating the access to a deeper area of the skin. These systems are also of interest for targeted therapy in the case of pathologies located in specific tissues [126].

### 3.6. Nanostructured Lipid Carriers

Nanoparticle-based formulations provide numerous advantages, such as long-term stability, improved patient compliance due to low cost, easy reproduction, and straightforward incorporation of both hydrophilic and hydrophobic active substances [189,190,191]. Nanostructured lipid carriers are more stable than solid lipid nanoparticles and can include more active substances [192,193]. Additionally, nanostructured lipid carriers facilitate the penetration of active substances into the deeper layers of the skin. They can include substances sensitive to external factors [194,195,196,197]. Also, lipid nanoparticles benefit from easily incorporating lipophilic substances that are poorly soluble in most solvents [198]. Formulations based on nanostructured lipid carriers demonstrate a prolonged and more significant release of active principles than those based on solid lipid nanoparticles. These formulations indicate a decrease in pore size, improved entrapment efficiency, and the release of active substances due to an increased lipophilic phase. Thus, nanostructured lipid-based formulations may constitute a viable option for the cutaneous release of MX [199].

## 4. Conclusions and Future Directions

**Scheme 2:**
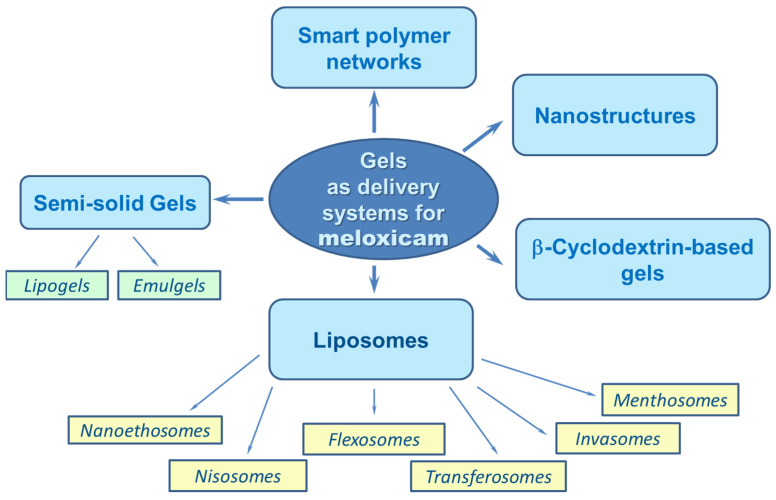
Types of gels used as delivery systems for meloxicam.

Meloxicam is an active substance with high potential as an anti-inflammatory and analgesic drug. Various drug delivery gels were designed over time to incorporate this hydrophobic drug, to extend the effective time of MX release, and to reduce the frequency of administration, thereby improving patient compliance and safety. Moreover, extensive studies have focused on enhancing the skin permeation of MX and a variety of drug carriers for transdermal MX delivery were developed: liposomes, semi-solid gels, nanostructures, or β-cyclodextrin-based gels (Scheme 2).

In recent years, significant advancements have been made in the field of transdermal drug delivery systems. However, each delivery system has advantages and drawbacks, as it was discussed recently [200,201]. All of the published studies suggest a bright perspective on the future development of transdermal MX-based carriers with controlled and targeted delivery and low adverse effects. The development of such systems allows for a better understanding of carrier effects on drug absorption and distribution through the skin, making them a promising tool for future pharmaceutical innovations. However, more in-depth research is needed regarding the interaction and distribution of the polymeric systems with/in the skin strata and to underline the drug-targeting mechanisms.

To overcome systemic side effects, it is essential to design and develop effective strategies for multifunctional carriers and targeted MX delivery systems, which can selectively target the inflamed joints. The major issues of the new tailored delivery systems are safety, efficacy, accessibility costs, and their use in clinical settings. In addition, they should be able to incorporate hydrophobic and lipophilic drugs, ensuring optimal bioavailability, controlled release, improved transdermal diffusion, and minimizing the side effects [202,203].

Another challenge for the near future can be the implementation of a theranostic approach for the management of inflammatory arthritis by designing smart stimuli-responsive biomaterials able to monitor the treatment status and then to suggest the most appropriate therapy for each patient [204,205]. Further investigation using novel technologies and non-invasive dosage forms for chronic disease therapy will be necessary to discover efficient administration techniques for various pharmaceutical forms.

## Figures and Tables

**Table 1:** The composition and some characteristics of various vesicular systems. Adapted with permission from [129] (copyright: 2020, Elsevier).

Vesicular System	Limitations	Particular Ingredients	Common Ingredients	Scalability
**Liposomes** Steady-state flux of 0.11 µg cm^−2^ h^−1^ [96]	Instability High cost [120,121] Rigidity [130,131] Easy degradation Instability during storage [132] Difficult to sterilize [133] Limited skin penetration [118] Possibility of oxidation Hydrolysis of phospholipids [134]	**Core** Aqueous drug solution	**Phospholipids** Phosphatidylcholine Phosphatidylserine Phosphatidylethanolamine Phosphatidylinositol **Stabilizer** Cholesterol	Certain well-defined production methods (extrusion, thin-film hydration) are reproducible, but meloxicam delivery systems have low scalability
**Ethosomes** Approx. 90% MX released within 7 h [135]	Skin irritation [136,137]	**Core** Hydroethanolic drug solution **Alcohols** Ethanol Isopropyl alcohol **Permeation enhancer** Propylene glycol **Charge Inducer** Stearylamine		Standard production methods ensure high scalability
**Transferosomes** Approx. 82% MX released from Carbopol-934 gel matrix in 24 h [11] Steady-state flux of 0.54 µg cm^−2^ h^−1^ [96]	Tend to accumulate within the skin [138] High cost Chemically unstable [139] Skin irritation	**Core** Aqueous drug solution **Edge activators** Sodium deoxycholate Sorbitan esters (Span 80/85) Dipotassium glycyrrhizinate Polysorbates		Complex production due to establishing an optimal ratio of surfactants, which requires higher costs
**Flexosomes**	Cutaneous irritation by the surfactant content High cost	**Core** Hydroethanolic drug solution **Alcohol** Ethanol **Edge activators** Tween 80 Sodium deoxycholate		Scalability depends on the complexity of the formulation
**Menthosomes**	Skin sensitization Physicochemical instability Possible interactions with other excipients due to menthol content	**Core** Aqueous menthol drug solution **Edge activators** Cationic surfactants(cetrimide) **Permeation enhancers** Menthol Cetylpyridinium chloride		Formulations need to be optimized for large-scale applications
**Invasomes**	Unstable over time [126,140] Cutaneous irritation [141,142]	**Core** Hydroethanolic terpene drug solution **Alcohol** Ethanol **Permeation enhancers** Terpenes: Limonene, Carvone, and Citral		Formulations need to be optimized for large-scale applications
**Niosomes** Good pharmacodynamic response [20]	Physical instability during storage [143,144]	**Core** Aqueous drug solution **Non-ionic surfactants** Polysorbates (Tween 20/40/60/80) Sorbitan esters (Span 20/40/60) Squalene Poloxamers Polyoxyethylene ethers		High scalability through extrusion, thin-film hydration [145]

## Data Availability

No new data were created or analyzed in this study.

## Abbreviations

The following abbreviations are used in this manuscript:

CMC	carboxymethyl cellulose
COX	cyclooxygenase enzyme
EC	ethyl cellulose
HEC	hydroxyethyl cellulose
HPC	hydroxypropyl cellulose
HPMC	hydroxypropyl methyl cellulose
HP-β-CD	hydroxypropyl-β-cyclodextrin
MX	meloxicam
NSAID	non-steroidal anti-inflammatory drug
PEG	poly(ethylene glycol)
PG	propylene glycol
PU	polyurethane
PVP	poly(vinyl pyrrolidone)
TEA	triethanolamine
β-CD	β-cyclodextrin
